# Why Cell-Free DNA Can Be a “Game Changer” for Lung Allograft Monitoring for Rejection and Infection

**DOI:** 10.1007/s13665-022-00292-8

**Published:** 2022-07-26

**Authors:** J.P. Rosenheck, B.C. Keller, G. Fehringer, Z.P. Demko, S.M. Bohrade, D.J. Ross

**Affiliations:** 1 Division of Pulmonary, Critical Care & Sleep Medicine, The Ohio State University, Columbus, OH USA; 2 Division of Pulmonary and Critical Care Medicine, Massachusetts General Hospital, Boston, MA USA; 3 Medical Affairs in Organ Health, Natera, Inc., San Carlos, USA; 4 Lung Transplant & Molecular Diagnostics, Natera, Inc, San Carlos, CA USA

**Keywords:** Lung transplant, Cell-free DNA, Donor-derived cell-free DNA, Biomarkers, Organ rejection

## Abstract

**Purpose of Review:**

Although there has been improvement in short-term clinical outcomes for patients following lung transplant (LT), advances have not translated into longer-term allograft survival. Furthermore, invasive biopsies are still standard of practice for monitoring LT recipients for allograft injury. We review the relevant literature supporting the role of using plasma donor-derived cell-free DNA (dd-cfDNA) as a non-invasive biomarker for LT allograft injury surveillance and discuss future research directions.

**Recent Findings:**

Accumulating data has demonstrated that dd-cfDNA is associated with molecular and cellular injury due to acute (cellular and antibody-mediated) rejection, chronic lung allograft dysfunction, and relevant infectious pathogens. Strong performance in distinguishing rejection and allograft injury from stable patients has set the stage for clinical trials to assess dd-cfDNA utility for surveillance of LT patients. Research investigating the potential role of dd-cfDNA methylation signatures to map injured tissue and cell-free DNA in detecting allograft injury-related pathogens is ongoing.

**Summary:**

There is an amassed breadth of clinical data to support a role for dd-cfDNA in monitoring rejection and other forms of allograft injury. Rigorously designed, robust clinical trials that encompass the diversity in patient demographics are paramount to furthering our understanding and adoption of plasma dd-cfDNA for surveillance of lung allograft health.

## Introduction

Lung transplant (LT) recipients are at risk for acute rejection (AR) and other allograft injuries that can lead to allograft failure. There is great interest in utilizing non-invasive biomarkers for early detection of rejection and allograft injury that would reduce the dependence on invasive biopsies for the surveillance of LT recipients. In this review, we discuss donor-derived cell-free DNA (dd-cfDNA) as a potential biomarker for monitoring allograft health in LT patients. We summarize the amassed data reporting elevated dd-cfDNA fraction associations with AR and other causes of allograft injury, along with test performance characteristics that support the launch of eagerly anticipated surveillance randomized-controlled trials. We also discuss future avenues of research in dd-cfDNA and cell-free DNA research.

## Lung Transplant and the Unmet Clinical Need for Non-invasive Biomarker Surveillance

Since the first human lung transplant by Dr. James Hardy at the University of Mississippi in 1963, the clinical indications for LT and surgical experience have vastly expanded [1]. The current data from the *Organ Procurement and Transplantation Network* (OPTN) reflect advances in the field with 3091 patients awaiting transplant and 2524 LT procedures performed in 2021, despite the impact of the COVID-19 pandemic on organ transplantation. New drug development has been a key driver of the increase in lung transplant volume over the past 50 years as immunosuppressive regimens have been refined with the advent of calcineurin and mTOR inhibitors. Post-transplant medical care has also evolved with an armamentarium of antibiotics, physiologic and roentgenographic surveillance, and fiberoptic bronchoscopy (FOB) techniques [2, 3].

Surveillance for acute rejection and other allograft injuries plays a critical role in lung allograft management. In a recent practice survey of 114 LT centers in 27 countries by the *International Society of Heart and Lung Transplant* (ISHLT), approximately 87% of centers performed surveillance FOB procedures. The current standard of practice (SOP) surveillance FOB includes both broncho-alveolar lavage (BAL) for microbiologic and cytologic studies and trans-bronchial biopsies (TBBx) for interrogation by histology [4]. A majority of centers perform surveillance procedures at 1, 3, 6, and 12 months [4].

Serious issues surround these procedures, and improvement in clinical outcomes and organ health has never convincingly been established [5]. Although histopathologic grading schemata for the diagnosis of acute rejection after LT have been well described, challenges persist with respect to inadequate alveolar tissue sampling and significant interobserver variability in pathologist reviews [6, 7]. In one analysis by Scott et al. approximately 18 biopsy specimens were deemed to be required to achieve 95% confidence for diagnosis of AR [8], a number unlikely to be obtained by most practitioners. Furthermore, serious adverse events such as pneumothorax, bronchial hemorrhage, hypoxemia, or acute respiratory failure associated with FOB and TBBx occur in roughly 10% of procedures [5, 9–11].

In a review of data from the ISHLT database, only modest improvement in 1- and 5-year survival rates in comparison with an earlier era (1996–2001) was observed, with a virtual plateau in clinical outcome measures during the most recent two decades [12]. Difficulties in confidently diagnosing AR hamper lung allograft management, contributing to the eventual development of chronic lung allograft dysfunction (CLAD), a pervasive and enigmatic cause of morbidity and mortality, that affects more than 50% of LT patients by 5 years post-transplant [12]. ISHLT data reveals that the incidence of CLAD mirrors that of LT survival rates, remaining unaltered over the last two decades [12].

Wisdom would dictate that earlier detection and treatment of complications such as AR and allograft infection may result in less morbidity and mortality and a decreased incidence of CLAD [13, 14]. Although our understanding of the pathobiology and risk factors for CLAD has broadened with time, no analyte in blood or bronchoalveolar lavage BAL fluid has yet been validated as a viable clinical biomarker [13, 15–19].

## Donor-Derived Cell-Free DNA and Assay Development

Circulating cell-free DNA (cfDNA) consists of short DNA fragments produced from nuclear and mitochondrial DNA, released during apoptosis, necrosis, or by active secretion from cells. The released cfDNA circulates bound to histones, protecting it from degradation by blood DNase. The majority of cfDNA is bound as mono-nucleosomes with a peak length of ~ 160 bp, which corresponds to the approximate length of DNA wrapped around a nucleosome, and a variable DNA linker fragment produced by apoptotic endonucleases [20, 21]. Recent evidence indicates that the majority of nucleosomal cfDNA is packaged into extracellular vesicles such as exosomes [22] or neutrophil extracellular traps (NETs) [23], with only a small fraction circulating as unbound nucleosomes. The biologic half-life of cfDNA is approximately 30 min to 2.5 h, with clearance predominantly mediated by the kidney and macrophage degradation in the liver and spleen [24].

From the standpoint of cfDNA, organ transplant is intriguingly analogous to the state of a singular pregnancy in that it is characterized by the presence of circulating cfDNA fragments representing two distinct genomes. Tissue injury incurred by an allograft organ is reflected by elevations in circulating levels of dd-cfDNA. Differentiation of donor from recipient cfDNA has been most recently accomplished by leveraging publicly available population genomic data to define a set of common and informative single nucleotide polymorphisms (SNPs) that can distinguish transplant donor-recipient pairs. This strategy is advantageous in that it requires only a recipient genetic sample (i.e., there is no need to collect donor samples for genomic sequencing). These techniques are now broadly applicable across racial and ethnically diverse patient populations and incorporated in commercially available assays [25, 26]. The methodologies for amplification and quantitation are variable and include whole-genome sequencing, targeted-genome sequencing, quantitative polymerase chain reaction (qPCR), and digital droplet polymerase chain reaction (ddPCR) [25, 26]. Typically, dd-cfDNA assays calculate the donor fraction of cfDNA (expressed as a percentage); however, absolute quantification (copies/mL) of dd-cfDNA may improve discrimination for active allograft rejection, particularly in the setting of increased background (recipient) genomic cfDNA observed in some patients, such as those with infection, high body mass index, recent surgery, or of older age [27–32].

## dd-cfDNA Entrée into the Lung Transplant Arena

In a pivotal investigation, De Vlaminck et al. implemented a genome transplant dynamics (GTD) shotgun sequencing SNP-based assay that requires pre-transplant whole-blood genome sequencing of both donor and recipient, to monitor for allograft injury. A total of 51 LT candidates were enrolled (bilateral = 44, unilateral = 7) and followed post-transplant, with an assessment of 398 longitudinal plasma samples. Post-LT, the dd-cfDNA fraction was initially elevated and decayed by double-exponential kinetics to a baseline by approximately 60 days. Data analysis of 113 paired TBBx that focused on the post 60-day time period, demonstrated an elevated dd-cfDNA fraction during moderate to severe (ISHLT ≥ grade A3) [7] acute cellular rejection (ACR) versus absence of ACR (grade A0) (*p* < 0.001). Using a ≥ 1.0% dd-cfDNA fraction cutoff, the sensitivity for AR was 100%, and the specificity was 73%. The area under the receiver operator characteristics curve (AUROC) was 0.90 (Table 1) [33]. dd-cfDNA fraction was also significantly elevated for antibody-mediated rejection (AMR) and (CLAD) relative to a quiescent group. When analyzing unilateral versus bilateral LT plasma samples in the absence of rejection, the cellular turnover rate was estimated as 58 and 107 cells/s, respectively, reflecting the inherently higher tissue mass associated with bilateral lung allografts. Henceforth, the investigators implemented a correction for unilateral LT samples by multiplying the dd-cfDNA fraction by a factor of 2 × prior to their analyses [33].

**Table 1:** Donor-derived cell-free DNA clinical validation studies in lung transplant with performance characteristics for allograft rejection

Study	Design	Patients (*N*)	Assay Type	dd-cfDNA threshold	SLT correction factor (2 ×)	Endpoint	Sensitivity/specificity	AUROC
De Vlaminck et al. [33]	Single-center, prospective cohort	51	Shotgun 2-genome genotyping	> 1.0%	Yes	ACR > A2	100%/73%	0.90
Sayah et al. [43]	Multi-center, prospective cohort	69	NGS targeted 206 SNPs	> 0.87%	No	ACR > A1	73%/53%	0.72
Khush et al. [44]	Single-center, prospective cohort	38	NGS targeted 206 SNPs	> 0.85%	No	ACR > A1 or AMR or BOS	56%/76%	0.67
Jang et al. [42••]	Multi-center, prospective cohort	148	Shotgun 2-genome genotyping	> 1.0%	Yes	ACR > A1 or AMR	77%/84%	0.89
Keller et al. [50•]	Multi-center, retrospective cohort	157	NGS targeted 206 SNPs	> 1.0%	No	ALAD	74%/88%* 67%/88%** 76%/70%***	0.82 0.79
Rosenheck et al. [45•]	Single-center, prospective cohort	103	mmPCR targeted 13,392 SNPs	> 1.0% > 1.0% > 1.0%	Yes Yes Yes	ACR > A1 or treated A1 or AMR ACR or AMR or CLAD ACR or AMR or CLAD or Infection	89%/83% 68%/84% 60%/84%	0.91 0.79 0.76

*NGS* next-generation sequencing, *dd-cfDNA* donor-derived cell-free DNA, *ACR* acute cellular rejection, *AMR* antibody-mediated rejection, *BOS* bronchiolitis obliterans syndrome, *CLAD* chronic lung allograft dysfunction, *SLT* single lung transplant, *AUROC* area under receiver operator characteristic curve, *SNPs* single nucleotide polymorphisms, *mmPCR* massively multiplexed polymerase chain reaction, *ALAD* acute lung allograft dysfunction (ACR + AMR + infection)

^*^Clinical diagnosis (not biopsy-proven) with 1-month follow-up as a criterion for stable cohort; **Clinical diagnosis (not biopsy-proven) with 3-month follow-up as a criterion for stable cohort; ***Biopsy-proven for surveillance criterion of stable cohort

Differences in dd-cfDNA between unilateral and bilateral LT recipients were further investigated in a recent analysis by the GRAfT consortium and NHLBI using two prospective cohort studies (221 patients) with serial plasma DNA measurement and contemporaneous TTBx and pulmonary function testing. The median dd-cfDNA fraction was lower for unilateral vs bilateral LT in stable controls (0.15%; interquartile range (IQR) 0.07–0.44% vs 0.46%; IQR 0.23–0.74%, *p* < 0.01) and AR (1.06%; IQR 0.75–2.32% vs 1.78%; IQR 1.18–5.73%, *p* = 0.05). Doubling dd-cfDNA for unilateral LT to account for the differences in lung mass eliminated this difference. Furthermore, the optimal dd-cfDNA threshold for the detection of AR without implementing a 2 × correction was 0.54% in unilateral LT and 1.1% in bilateral LT [34•]. The *Genomic Research Alliance for Transplantation* (GRAfT), a consortium of NHLBI thoracic transplant centers, has further assessed dd-cfDNA clinical validity and utility in the LT population. Using the GTD shotgun technique, Agbor-Enoh and colleagues at NIH analyzed dd-cfDNA fraction for longitudinal plasma samples obtained 14–90 days post-transplant from 106 LT recipients. The highest tertile for median dd-cfDNA fraction, when compared to the lowest dd-cfDNA fraction tertile, was associated with a 6.6-fold increased risk for later development of allograft failure due to CLAD [35]. This finding that early temporal events characterized by intra-graft inflammation and injury can predict the latter development of CLAD is further supported by studies of inflammatory chemokines such as CCR2 ligand, CXCR3 ligand, CXCL10 ligand, and TGFß [36–39]. An intriguing investigation by Yang et al. measured CXCL10 (by ELISA) and cfDNA in 60 BAL samples with either obstructive or restrictive CLAD or designated as stable. An association of both analytes with overall allograft survival was observed with an interaction between cfDNA and CXCL10 [39].

In a further collaborative study by GRAfT, the researchers evaluated the kinetics of dd-cfDNA for episodes of ACR and AMR as adjudicated by ISHLT accepted criteria [7, 40]. Episodes of AMR were associated with more tissue injury as determined by greater spirometric decline and higher dd-cfDNA fraction compared to ACR episodes (5.4% vs 1.1%, *p* < 0.01). Significantly, evidence for allograft injury as detected by dd-cfDNA, preceded clinical AMR diagnosis as determined by standard spirometry and histopathology by a median of 2.8 months [41], whereas elevations in dd-cfDNA fraction occurred nearly simultaneously with the onset of ACR.

In a prospective multi-center cohort study in GRAfT, Jang et al. analyzed longitudinal dd-cfDNA samples associated with contemporaneous TBBx procedures in 148 patients over a median of 19.6 months after LT [42••]. For AR, the dd-cfDNA fraction was elevated six-fold compared to controls. dd-cfDNA fraction also correlated with the severity of spirometric decline and histological grading of AR. The dd-cfDNA fraction AUROC for AR, AMR, and ACR was 0.89, 0.93, and 0.83, respectively. Furthermore, standard histopathology only detected one-third of episodes when the dd-cfDNA fraction was ≥ 1.0%, while > 90% of these episodes were coincident with clinical complications missed on TBBx. This finding underscores the vexing dilemma of comparing the performance characteristics of dd-cfDNA to a potentially flawed touchstone of standard histopathology [42••].

## Commercially Available Tests for Plasma dd-cfDNA Validated in Lung Transplant

**Fig. 1:**
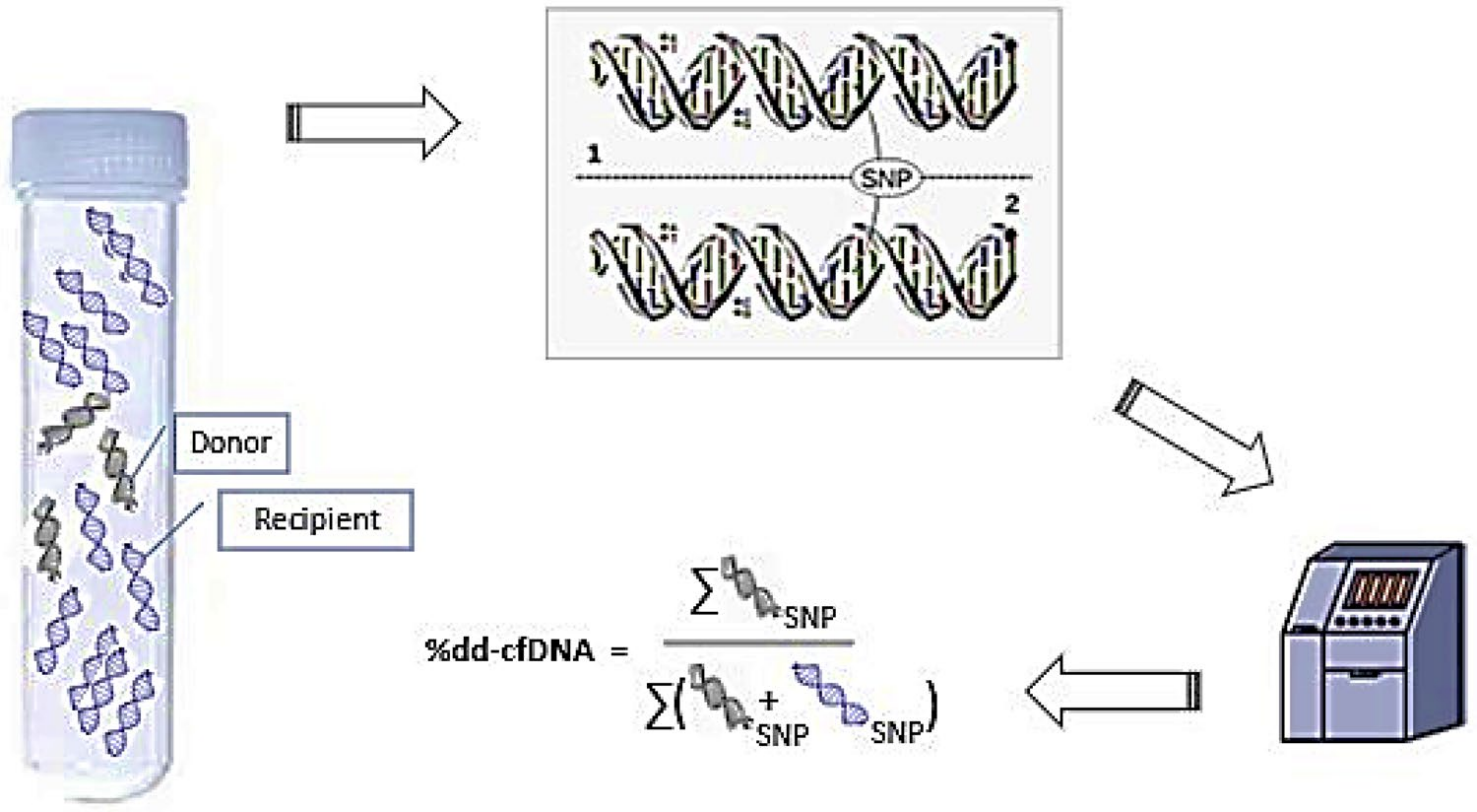
Commercial tests to quantify the fraction of donor-derived cell-free DNA. There are approximately 4–5 million SNPs in any individual while more than 100 million SNPs span populations worldwide [67]. Commercially available tests now quantify donor fraction dd-cfDNA from a single recipient plasma sample by leveraging disparities in single nucleotide polymorphisms (SNPs) between donor and recipient with complex bioinformatic algorithms. Quantification utilizes either targeted next-generation sequencing (NGS), massively complexed polymerase chain reaction (mmPCR), or digital droplet PCR (ddPCR). The donor fraction (%) represents donor relative to total background (donor + recipient) cfDNA

Laboratory-developed tests (LDT) which are performed in accordance with the *Clinical Laboratory Improvement Amendments of 1988* (CLIA)—certification by the Centers for Medicare & Medicaid Services CMS and *The College of American Pathologists* (CAP)—are now commercially available for clinical dd-cfDNA testing after LT. Test methodology, analytical validation, threshold, validation study population, and performance characteristics have varied among assays (Table 1) [25, 26, 43, 44, 45•]. Available commercial tests, however, all leverage disparities in curated panels of SNPs to quantify dd-cfDNA fraction (Fig. 1).

### Prospera™

Prospera™ is a commercially available LDT performed in a CLIA-certified and CAP-approved commercial laboratory (Natera, Inc.; Austin, TX, USA). dd-cfDNA fraction is determined with a massively multiplexed polymerase chain reaction (mmPCR) assay for a curated panel of over 13,000 SNPs across all ethnicities with a lower limit for quantitation of 0.05% [25]. In a recent study, investigators at The Ohio State University prospectively collected 195 plasma samples (103 patients) with adjudicated clinical-pathologic diagnoses while blinded to results of concurrent dd-cfDNA fraction [45•]. The dd-cfDNA fraction determined by the Prospera test was compared across clinical-pathologic cohorts: stable, ACR, AMR, isolated lymphocytic bronchiolitis (ISHLT grade B), CLAD/neutrophilic-responsive allograft dysfunction (CLAD/NRAD), and infection. Median dd-cfDNA fraction was significantly higher for ACR (1.43%; IQR 0.67–2.32%, *p* < 0.001), AMR (2.50%; IQR 2.06–3.79%, *p* < 0.001), allograft infection (0.74%; IQR 0.46–1.38%, *p* = 0.02), and CLAD/NRAD (1.60%; IQR 0.57–2.60%, *p* < 0.001) relative to the stable cohort. The AUROC for AR versus stable was 0.91 (95% CI 0.83–0.98). Using a ≥ 1% dd-cfDNA fraction threshold, the sensitivity for AR was 89.1% (95% CI 76.2–100.0%), specificity 82.9% (95% CI 73.3–92.4%), PPV 51.9% (95% CI 37.5–66.3%), and negative predictive value (NPV) 97.3% (95% CI 94.3–100.0%) based on the study cohort prevalence for AR of 17.2% (Table 1). For combined allograft injury (ACR + AMR + CLAD/NRAD + infection), the AUROC was 0.76 (95% CI 0.66–0.85%), sensitivity 59.9% (95% CI 46.0–73.9%), and specificity 83.9% (95% CI 74.1–93.7%). The investigators further analyzed the differences in dd-cfDNA fractions in single lung transplant (SLT) and bilateral lung transplant (BLT) patients in stable and AR cohorts. The median dd-cfDNA fraction in the stable cohort was 2.7-fold higher for BLT (0.56%; IQR 0.31–0.87%, *n* = 76) than for SLT (0.21%; IQR 0.11–0.37%, *n* = 23) (*p* < 0.001). The median dd-cfDNA fraction for the 4 episodes of AR in the SLT group (0.74%; IQR 0.19–1.31%, *n* = 4) was lower than that in the BLT group (1.98%; IQR 1.10–2.87%, *n* = 31). The authors recommended instituting a 2 × multiplier correction factor for dd-cfDNA fraction obtained from SLT patients based on their data and results from other studies [33, 35, 42••, 46, 47].

Prospera™ will be further investigated in two studies. Lung Allograft Monitoring with Blood Dd-cfDNA Assessments (LAMBDA 001) is a multi-national, non-inferiority, parallel-design, randomized-controlled trial of SOP surveillance TBBx procedures versus plasma dd-cfDNA monitoring during the initial 1-year after LT with a primary endpoint of hospital-free days. The study is being conducted through a Clinical Trial Agreement between the *National Heart, Lung, and Blood Institute* (NHLBI) and Natera, Inc. (San Antonio, TX, USA). The study sample size of 400 new LT patients allocated to treatment or control arms, provides a power > 95% for non-inferiority or superiority of dd-cfDNA monitoring. Secondary endpoints include severe allograft infection, FEV1 (forced expiratory volume in one second) at 12 months, CLAD, allograft failure, de novo donor-specific antibodies (DSA), and all-cause mortality. Plasma will also be analyzed for metagenomics, epigenetic signatures, and absolute quantification of dd-cfDNA. LAMBDA 002, an observational cohort study, will investigate the development of CLAD at 3-years after LT in the longer-term follow-up of the LAMBDA 001 cohorts, in addition to approximately 600 non-randomized patients less than 1-year after LT. A sub-study will explore the additional value of tissue gene expression profiling (GEP) of TBBx specimens.

### AlloSure™

Another commercially available LDT, AlloSure™ (CareDx, Inc.; Brisbane, CA, USA), implements targeted amplification and next-generation sequencing (NGS) in a clinical-grade assay with a panel of 266 SNPs selected based on allele frequency across ancestral heritage groups, sequencing accuracy, and lack of linkage. The dd-cfDNA assay, AlloSure™v1.0, measures dd-cfDNA with a lower limit of detection of 0.16% and limit of quantification of 0.20% [26]. The assay has recently been updated (AlloSure™ v3.0) with 405 SNPs and a reportable range of detection from 0.12 to 16.0% [48]. From 69 archival plasma samples (one sample per patient) obtained during the Lung Allograft Rejection Gene Expression Observational (LARGO) study (ClinicalTrials.gov Identifier: NCT00751309; initiated in April 2004), Sayah et al. analyzed AlloSure™ v1.0 dd-cfDNA and paired TBBx histopathology adjudicated reports. The median dd-cfDNA fraction in ACR patients was significantly elevated (1.52%; IQR 0.52–2.26%) compared with normal stable patients (0.49%; IQR 0.22–0.79%) (*p* = 0.03). dd-cfDNA values were not different for the infection group (0.60%; IQR 0.27–1.17%) relative to the normal (*p* = 0.28) and ACR (*p* = 0.10) groups. The AUROC for ACR versus the stable group was 0.72 (95% CI 0.55–0.89) (*p* = 0.03). At an optimal threshold of 0.87% dd-cfDNA fraction, the sensitivity for ACR was 73.1%, specificity 52.9%, positive predictive value (PPV) 34.1%, and NPV 85.5% assuming a prevalence of 25% (Table 1) [43]. The performance for dd-cfDNA comparing a combined injury group to stable patients was not reported. A criticism of this study related to uncertainty regarding the reliability of histopathologic diagnosis in LARGO, as further review of 1566 TBBx from 845 subjects yielded a suboptimal kappa value for interobserver agreement (0.18; 95% CI 0.15–0.22) (*p* < 0.001) [49].

Khush et al. further evaluated biorepository samples derived from the original Stanford GTD study, measuring AlloSure™ v1.0 dd-cfDNA fraction in 107 plasma samples from 38 unique LT recipients with defined diagnostic cohorts classified as either biopsy-confirmed or treated ACR, AMR, obstructive CLAD, allograft infection, and stable allografts. The median dd-cfDNA fraction was elevated in ACR (0.91%; IQR 0.39–2.07%), CLAD (2.06%; IQR 0.57–3.67%), and an aggregated cohort of rejection (combined ACR, AMR, and CLAD) (1.06%; IQR 0.38–2.51%), in comparison with the stable cohort (0.38%; IQR 0.23–0.87%) (*p* = 0.02). The dd-cfDNA fraction for the AMR cohort was elevated (1.34%; IQR 0.34–2.40%) compared to the stable cohort, although the result was not statistically significant (*p* = 0.07). No difference in the median dd-cfDNA fraction for allograft infection (0.39%; IQR 0.18–0.67%) versus the stable group was found, which the authors speculated might be related to difficulty in distinguishing infection from airway colonization. In contrast to the original analysis by De Vlaminck et al. there was no apparent difference in dd-cfDNA fraction for unilateral versus bilateral LT samples [33]. The determined optimal threshold for dd-cfDNA fraction for aggregated rejection was 0.85% which yielded a sensitivity of 55.6%, specificity 75.8%, PPV 43.3%, NPV 83.6%, and AUROC of 0.67 (95% CI 0.59–0.74) (Table 1) [44].

During the early era of the COVID-19 global pandemic (March 24, 2020, to September 01, 2020), a consortium of 4 LT centers implemented dd-cfDNA (AlloSure) surveillance testing in addition to each center’s SOP and telehealth visits. Due to COVID-19 contagion risks, SOP surveillance TBBx at these centers was suspended and only for cause procedures were performed. In a retrospective analysis of patient chart reviews at these centers, Keller et al. described the performance characteristics for remote monitoring with dd-cfDNA (Allosure) [50•]. A total of 290 samples from 157 LT patients between 1 month and 3 years after transplant were included in the analysis. Median dd-cfDNA fraction was 1.7% (IQR 0.63–3.1%) for the composite endpoint of acute lung allograft dysfunction (ALAD) that encompassed ACR, AMR, and allograft infection versus 0.35% (IQR 0.22–0.79%; *p* < 0.001) for a stable cohort. Since TBBx procedures were limited, assignment to the stable cohort was based on stability in-home spirometry FEV1 for 1 month following the AlloSure test and the absence of diagnosed ACR, AMR, and infection. In total, 82% of dd-cfDNA samples did not have the accepted gold standard surveillance TBBx for the assessment of ACR or confirmation of stable cohort assignment. No adjustments for SLT samples were performed during the study design, although analyses demonstrated that fractions were statistically lower for SLT vs BLT for stable samples. For allograft infection, the median dd-cfDNA fraction was elevated (1.8%; IQR 0.84–2.7%) compared to the stable cohort but not different from the AR cohort (*p* = 0.82). The sensitivity, specificity, PPV, and NPV for distinguishing ALAD from the stable cohort was 74% (95% CI 54–87%), 88% (95% CI 82–92%), 43% (95% CI 31–57%), and 97% (95% CI 93–98%), respectively. The AUROC was 0.82. The performance was 76% (95% CI 54–90%), 70% (95% CI 51–85%), 67% (95% CI 48–81), and 79% (95% CI 59–91%), respectively, for *N* = 52 samples where SOP surveillance TBBx were paired with samples with measured dd-cfDNA fraction (Table 1). Despite the limitation of frequently missing TBBx data, this study provided real-world evidence that highlighted the potential value of dd-cfDNA monitoring and telehealth during the COVID-19 pandemic [50•].

Further data for AlloSure™ is forthcoming, with a multi-center US observational registry, AlloSure Lung Assessment and Metagenomics Outcomes Study (ALAMO; ClinicalTrials.gov Identifier: NCT05050955) assessing the value of multi-modality molecular tools in plasma and tissue.

## Medical Economics and Lung dd-cfDNA Scheduled Testing

No medical economic analysis has yet been published regarding the impact of %dd-cfDNA monitoring versus SOP surveillance FOB with TBBx assessments after LT. Although an imperfect comparison, a review of costs related to surveillance procedures in lung cancer screening reveals significant costs related to the minor (median $5573), intermediate (median $19,470), and major (median $57,893) complications [51] (reported for the 65–69 year age group).

These potential costs can be considered in the context of the complication rates and benefits from TBBx. In a 1-year comprehensive analysis of bronchoscopy surveillance after LT, McWilliams et al. reported that complication rates for the time after LT of 0–3 months, 3–12 months, and > 12 months were 25.7%, 18.9%, and 24.3%, respectively. Overall, the pneumothorax rate was approximately 2%, while the majority of complications related to sedation or endobronchial hemorrhage. These complication rates suggest substantial indirect costs are associated with surveillance TBBx. Compounding the risks associated with surveillance transbronchial biopsies is that of insufficient tissue for adequate histopathologic^7^ review, observed in 5.6% of procedures, similar in magnitude to the rate of significant pathological findings found in only 21.6% of surveillance TBBx [7, 52]. Other studies also highlight the limitations in TBBx. For example, in a study by Levy et al. AX (insufficient for grading) surveillance biopsies in clinically stable patients were not treated with augmented immunosuppression and not re-biopsied. Despite this, no increased risk of CLAD, retransplant, or death was observed [53].

Internal modeling comparing the use of cfDNA in place of surveillance biopsy for lung transplant rejection indicates the potential for greater than 1 million dollars of net savings per 100 patient-years, even when accounting for the expected false-positive rates of this testing strategy. Currently, no supporting data has been published; however, confirmation of this medical model is anticipated in future prospective randomized controlled trials such as LAMBDA 001.

## Future Directions

### Metagenomics and Next-Generation Sequencing of Plasma Cell-Free DNA and RNA

Considerable interest has developed in the potential utility of untargeted metagenomic next-generation sequencing (mNGS) analysis of plasma cell-free DNA and RNA for the detection of microbial pathogens (bacteria, fungi, viruses, parasites). Targeted nucleic acid amplification tests (NAAT) utilize polymerase chain reaction (PCR) primers for clinically suspected microbial pathogens; however, NGS provides the opportunity for testing using an expansive panel (> 1000) of potential pathogens to identify novel associations with allograft injury that can lead to the identification of clinically significant pathogens.

A limitation of mNGS is that the sensitivity is critically dependent on the level of background human cfDNA relative to microbial DNA and RNA. Therefore, commercially available LDT for plasma metagenomics employs methodology to eliminate human cfDNA signatures from plasma, enhanced sequencing read depth, and bioinformatics to detect the minute fraction representing microbial DNA and RNA [54]. Further research and clinical data are required to eliminate potential sources of microbial contamination in these highly sensitive tests and to determine thresholds for differentiation of clinically significant pathogens in plasma from background microbial DNA that may be normal commensal [55, 56].

Host gene expression ensuant to infection is another intriguing application of mNGS technology. RNA libraries derived from mNGS for the detection of pathogens such as RNA viruses incidentally produce host and microbial gene expression data for transcriptome (RNA-seq) analyses [57]. Although, to date, no cfRNA-seq–based assay has been clinically validated for use in patients, the potential clinical impact of cfRNA-seq analyses could be significant. Interrogation of cfRNA corresponding to active microbial gene expression might enable the discrimination between infection versus colonization [54] and differentiation of viable from non-viable organisms that do not impact organ health [58]. This technology may also facilitate the identification of potential resistance markers to antimicrobial therapies. Moreover, cfRNA-seq analyses of the human host and microbial pathogens can be used to identify novel or underappreciated host–microbial interactions directly from clinical samples [59].

### Epigenetic Signatures May Complement dd-cfDNA

Differentiation of tissue injury pathogenesis due to ACR, AMR, CLAD, and infection would add great value to the non-invasive diagnosis for complications of transplant. Currently, dd-cfDNA represents a biomarker of injury but usually requires diagnostic FOB with BAL microbiology and TBBx to ascertain a specific clinical diagnosis.

Since cfDNA maintains the methylation signatures of its tissue of origin, cfDNA methylomic analysis by bisulfite sequencing can enable mapping of injured tissue and add further understanding to the detected dd-cfDNA fraction [60–62]. Indeed, in a recent study of COVID-19 patients, investigators interrogated cfDNA methylation signatures to define the afflicted tissues associated with the SARS-CoV-2 infection [61]. The delineation of methylation signatures may provide further insights into the origin of dd-cfDNA elaboration and help differentiate unique phenotypes of rejection [61, 63, 64].

Furthermore, epigenetic modulation of immune system-related gene expression can dynamically regulate innate and adaptive immune responses and ultimately influence allograft survival [65]. In a kidney transplant investigation by Zhu et al. AR-induced allograft dysfunction was associated with changes in hypermethylation of peripheral blood mononuclear cells of allograft recipients. Pathway enrichment analysis of the differentially methylated regions disclosed hypermethylated genes that were primarily involved in immune-related signaling pathways (including the mTOR pathway) [66]. Therefore, methylomic analyses may provide enhanced mechanistic insights into allograft dysfunction and immune regulation that translate into individualized treatment algorithms.

### Donor-Derived Cell-Free DNA, Not Only a Biomarker but also a Provocateur of Tissue Injury?

Emerging evidence suggests that cfDNA and mitochondrial cfDNA (mtcfDNA) can directly elicit tissue injury by acting as damage-associated molecular patterns (DAMPs) through pattern recognition receptors (PRRs) such as toll-like receptor 9 (TLR9), hence activating innate immune response or cell death pathways [64]. If true, this mechanism may provide a new pathway to implement novel therapeutic strategies, incorporating scavenging or otherwise mitigating the effect of tissue-specific cfDNA through PRR blockade [64]. Furthermore, data supporting an association between dd-cfDNA and CLAD is also consonant with both murine and human findings for a putative role for NETs and associated cfDNA in the pathogenesis of CLAD [23], although the relationship between NETs, cfDNA, and allograft injury requires further clarification.

## Conclusions

There has long been an unmet need for non-invasive tools for surveillance of the lung transplant population, recently underscored by the ongoing COVID-19 pandemic. Although plasma dd-cfDNA monitoring is in a nascent stage of its evolution and utility, considerable optimism is afforded by an amassed breadth of clinical data to support its utility as a form of precision medicine. Rigorously designed, robust clinical trials that encompass the diversity in patient demographics are paramount to furthering our understanding and adoption of plasma dd-cfDNA for surveillance of lung allograft health. There is considerable promise for cfDNA as a potential biomarker and endpoint for future clinical trial development. Furthermore, the putative indirect effects of dd-cfDNA on allograft injury via innate and adaptive immune responses are an intriguing area for future scientific discovery.

## Abbreviations

ACR: Acute cellular rejection
ALAD: Acute lung allograft dysfunction
AMR: Antibody-mediated rejection
AUROC: Area under the receiver operator characteristics curve
BAL: Bronchoalveolar lavage
BLT: Bilateral lung transplant
CAP: College of American Pathologists
CLAD: Chronic lung allograft dysfunction
FEV1: Forced expiratory volume in 1 s
GTD: Genome transplant dynamics
IQR: Interquartile range
ISHLT: International Society of Heart and Lung Transplant
LAMBDA: Lung Allograft Monitoring with Blood Dd-cfDNA Assessments Study
LARGO: Lung Allograft Rejection Gene Expression Observational Study
LDT: Laboratory-developed tests
LT: Lung transplant
NETs: Neutrophil extracellular traps
NGS: Next-generation sequencing
NHLBI: National Heart, Lung, and Blood Institute
NPV: Negative predictive value
NRAD: Neutrophilic-responsive allograft dysfunction
PCR: Polymerase chain reaction
PRR: Pattern recognition receptors
SLT: Single lung transplant
SNP: Single nucleotide polymorphisms
SOP: Standard of practice
TBBx: Trans-bronchial biopsies

## References

[1] Hardy JD, Webb WR, Dalton ML, Walker GR (1963). Lung homotransplantation in man: report of the initial case. JAMA.

[2] Chandrashekaran S, Crow Pharm SA, Shah SZ, Arendt Pharm CJ, Kennedy CC (2018). Immunosuppression for lung transplantation: current and future. Curr Transplant Rep.

[3] Guilinger RA, Paradis IL, Dauber JH, Yousem SA, Williams PA, Keenan RJ (1995). The importance of bronchoscopy with transbronchial biopsy and bronchoalveolar lavage in the management of lung transplant recipients. Am J Respir Crit Care Med.

[4] Martinu T, Koutsokera A, Benden C, Cantu E, Chambers D, Cypel M (2020). International Society for Heart and Lung Transplantation consensus statement for the standardization of bronchoalveolar lavage in lung transplantation. J Heart Lung Transplant.

[5] Valentine VG, Gupta MR, Weill D, Lombard GA, LaPlace SG, Seoane L (2009). Single-institution study evaluating the utility of surveillance bronchoscopy after lung transplantation. J Heart Lung Transplant.

[6] Bhorade SM, Husain AN, Liao C, Li LC, Ahya VN, Baz MA (2013). Interobserver variability in grading transbronchial lung biopsy specimens after lung transplantation. Chest.

[7] Stewart S, Fishbein MC, Snell GI, Berry GJ, Boehler A, Burke MM (2007). Revision of the 1996 working formulation for the standardization of nomenclature in the diagnosis of lung rejection. J Heart Lung Transplant.

[8] Scott JP, Fradet G, Smyth RL, Mullins P, Pratt A, Clelland CA, et al. Prospective study of transbronchial biopsies in the management of heart-lung and single lung transplant patients. J Heart Lung Transplant. 1991;10(5 Pt 1):626–36; discussion 36–7. 1958673

[9] Chhajed PN, Aboyoun C, Malouf MA, Hopkins PM, Plit ML, Glanville AR (2003). Risk factors and management of bleeding associated with transbronchial lung biopsy in lung transplant recipients. J Heart Lung Transplant.

[10] Hopkins PM, Aboyoun CL, Chhajed PN, Malouf MA, Plit ML, Rainer SP (2002). Prospective analysis of 1,235 transbronchial lung biopsies in lung transplant recipients. J Heart Lung Transplant.

[11] Vitulo P, Cremaschi P, Arbustini E, Volpato G, Volpini E, Martinelli L (1996). Surveillance transbronchial biopsy in the diagnosis of acute lung rejection in heart and lung and lung transplant recipients. Monaldi Arch Chest Dis.

[12] Chambers DC, Perch M, Zuckermann A, Cherikh WS, Harhay MO, HayesJr D (2021). The international thoracic organ transplant registry of the international society for heart and lung transplantation: thirty-eighth adult lung transplantation report - 2021; Focus on recipient characteristics. J Heart Lung Transplant.

[13] Verleden GM, Glanville AR, Lease ED, Fisher AJ, Calabrese F, Corris PA (2019). Chronic lung allograft dysfunction: definition, diagnostic criteria, and approaches to treatment-a consensus report from the Pulmonary Council of the ISHLT. J Heart Lung Transplant.

[14] Amubieya O, Ramsey A, DerHovanessian A, Fishbein GA, Lynch JP, Belperio JA (2021). Chronic lung allograft dysfunction: evolving concepts and therapies. Semin Respir Crit Care Med.

[15] Glanville AR, Verleden GM, Todd JL, Benden C, Calabrese F, Gottlieb J (2019). Chronic lung allograft dysfunction: definition and update of restrictive allograft syndrome-a consensus report from the Pulmonary Council of the ISHLT. J Heart Lung Transplant.

[16] Shino MY, DerHovanessian A, Sayah DM, Saggar R, Ying Xue Y, Ardehali A, et al. The impact of allograft CXCL9 during respiratory infection on the risk of chronic lung allograft dysfunction. OBM Transplant. 2018;2(4). 10.21926/obm.transplant.1804029 PMC6693350 31414076

[17] Shino MY, Weigt SS, Li N, Derhovanessian A, Sayah DM, Saggar R (2018). The prognostic importance of bronchoalveolar lavage fluid CXCL9 during minimal acute rejection on the risk of chronic lung allograft dysfunction. Am J Transplant.

[18] Shino MY, Weigt SS, Li N, Palchevskiy V, Derhovanessian A, Saggar R (2017). The prognostic importance of CXCR3 chemokine during organizing pneumonia on the risk of chronic lung allograft dysfunction after lung transplantation. PLoS ONE.

[19] Shino MY, Zhang Q, Li N, Derhovanessian A, Ramsey A, Saggar R (2021). The allograft injury marker CXCL9 determines prognosis of anti-HLA antibodies after lung transplantation. Am J Transplant.

[20] Heitzer E, Auinger L, Speicher MR (2020). Cell-free DNA and apoptosis: how dead cells inform about the living. Trends Mol Med.

[21] Chiu RWK, Heitzer E, Lo YMD, Mouliere F, Tsui DWY (2020). Cell-free DNA fragmentomics: the new “omics” on the block. Clin Chem.

[22] Fernando MR, Jiang C, Krzyzanowski GD, Ryan WL (2017). New evidence that a large proportion of human blood plasma cell-free DNA is localized in exosomes. PLoS ONE.

[23] Sayah DM, Mallavia B, Liu F, Ortiz-Munoz G, Caudrillier A, DerHovanessian A (2015). Neutrophil extracellular traps are pathogenic in primary graft dysfunction after lung transplantation. Am J Respir Crit Care Med.

[24] Yao W, Mei C, Nan X, Hui L (2016). Evaluation and comparison of in vitro degradation kinetics of DNA in serum, urine and saliva: a qualitative study. Gene.

[25] Altug Y, Liang N, Ram R, Ravi H, Ahmed E, Brevnov M (2019). Analytical validation of a single-nucleotide polymorphism-based donor-derived cell-free DNA assay for detecting rejection in kidney transplant patients. Transplantation.

[26] Grskovic M, Hiller DJ, Eubank LA, Sninsky JJ, Christopherson C, Collins JP (2016). Validation of a clinical-grade assay to measure donor-derived cell-free DNA in solid organ transplant recipients. J Mol Diagn.

[27] Bunnapradist S, Homkrailas P, Ahmed E, Fehringer G, Billings PR, Tabriziani H (2021). Using both the fraction and quantity of donor-derived cell-free DNA to detect kidney allograft rejection. J Am Soc Nephrol.

[28] Oellerich M, Shipkova M, Asendorf T, Walson PD, Schauerte V, Mettenmeyer N (2019). Absolute quantification of donor-derived cell-free DNA as a marker of rejection and graft injury in kidney transplantation: results from a prospective observational study. Am J Transplant.

[29] Osmanodja B, Akifova A, Budde K, Choi M, Oellerich M, Schutz E (2021). Absolute or relative quantification of donor-derived cell-free DNA in kidney transplant recipients: case series. Transplant Direct.

[30] Qi Y, Uchida T, Yamamoto M, Yamamoto Y, Kido K, Ito H (2016). Perioperative elevation in cell-free DNA levels in patients undergoing cardiac surgery: possible contribution of neutrophil extracellular traps to perioperative renal dysfunction. Anesthesiol Res Pract.

[31] Meddeb R, Dache ZAA, Thezenas S, Otandault A, Tanos R, Pastor B (2019). Quantifying circulating cell-free DNA in humans. Sci Rep.

[32] Vora NL, Johnson KL, Basu S, Catalano PM, Hauguel-De Mouzon S, Bianchi DW (2012). A multifactorial relationship exists between total circulating cell-free DNA levels and maternal BMI. Prenat Diagn.

[33] De Vlaminck I, Martin L, Kertesz M, Patel K, Kowarsky M, Strehl C (2015). Noninvasive monitoring of infection and rejection after lung transplantation. Proc Natl Acad Sci U S A.

[34] • Keller MB, Meda R, Fu S, Yu K, Jang MK, Charya A, et al. Comparison of donor-derived cell-free DNA between single vs. double lung transplant recipients. Am J Transplant. 2022. 10.1111/ajt.17039. Analysis of accumulated data from the GRAfT investigators demonstrating higher dd-cfDNA fractions for double lung versus single lung transplant recipients. **The analysis confirmed an algorithm previously implemented by these investigators, multiplying single lung transplant dd-cfDNA levels x 2 for interpretation against a threshold dd-cfDNA level.**

[35] Agbor-Enoh S, Wang Y, Tunc I, Jang MK, Davis A, De Vlaminck I (2019). Donor-derived cell-free DNA predicts allograft failure and mortality after lung transplantation. EBioMedicine.

[36] Der Hovanessian A, Palchevskiy V, Weigt SS, Shino MY, Gregson AL, Kubak BM, et al. The CCL2/CCR2 axis in primary graft dysfunction and bronchiolitis obliterans syndrome following lung transplantation. J Heart Lung Transplant. 2013;32(4, Supplement):S28-S9.

[37] DerHovanessian A, Weigt SS, Palchevskiy V, Shino MY, Sayah DM, Gregson AL (2016). The role of TGF-β in the association between primary graft dysfunction and bronchiolitis obliterans syndrome. Am J Transplant Off J Am Soc Transplant Am Soc Transplant Surg.

[38] Shino MY, Weigt SS, Li N, Palchevskiy V, Derhovanessian A, Saggar R (2013). CXCR3 ligands are associated with the continuum of diffuse alveolar damage to chronic lung allograft dysfunction. Am J Respir Crit Care Med.

[39] Yang JYC, Verleden SE, Zarinsefat A, Vanaudenaerde BM, Vos R, Verleden GM (2019). Cell-free DNA and CXCL10 derived from bronchoalveolar lavage predict lung transplant survival. J Clin Med.

[40] Levine DJ, Glanville AR, Aboyoun C, Belperio J, Benden C, Berry GJ (2016). Antibody-mediated rejection of the lung: a consensus report of the International Society for Heart and Lung Transplantation. J Heart Lung Transplant.

[41] Agbor-Enoh S, Jackson AM, Tunc I, Berry GJ, Cochrane A, Grimm D (2018). Late manifestation of alloantibody-associated injury and clinical pulmonary antibody-mediated rejection: evidence from cell-free DNA analysis. J Heart Lung Transplant.

[42] •• Jang MK, Tunc I, Berry GJ, Marboe C, Kong H, Keller MB, et al. Donor-derived cell-free DNA accurately detects acute rejection in lung transplant patients, a multicenter cohort study. J Heart Lung Transplant. 2021;40(8):822–30. **An in depth analysis of dd-cfDNA test performance in a well-designed multicenter observational cohort study by the GRAfT investigators.** 10.1016/j.healun.2021.04.009 PMC8319066 34130911

[43] Sayah D, Weigt SS, Ramsey A, Ardehali A, Golden J, Ross DJ (2020). Plasma donor-derived cell-free DNA levels are increased during acute cellular rejection after lung transplant: pilot data. Transplant Direct.

[44] Khush KK, De Vlaminck I, Luikart H, Ross DJ, Nicolls MR. Donor-derived, cell-free DNA levels by next-generation targeted sequencing are elevated in allograft rejection after lung transplantation. ERJ Open Res. 2021;7(1). :00462–2020. 10.1183/23120541.00462-2020 PMC7836440 33532456

[45] • Rosenheck JP, Ross DJ, Botros M, Wong A, Sternberg J, Chen Y-A, et al. Clinical validation of a plasma donor-derived cell-free DNA assay to detect allograft rejection and injury in lung transplant. Transplantation Direct. 2022;8(4). 10.1097/TXD.0000000000001317. **Pivotal data implementing a clinically-available dd-cfDNA assay in a blinded, cohort study of ACR, AMR, CLAD, and allograft infection in advance of development of a noninferiority-design surveillance. randomized-controlled multi-center trial.** 10.1097/TXD.0000000000001317 PMC8963832 35372675

[46] Keller M, Bush E, Diamond JM, Shah P, Matthew J, Brown AW (2021). Use of donor-derived-cell-free DNA as a marker of early allograft injury in primary graft dysfunction (PGD) to predict the risk of chronic lung allograft dysfunction (CLAD). J Heart Lung Transplant.

[47] Bazemore K, Rohly M, Permpalung N, Yu K, Timofte I, Brown AW (2021). Donor derived cell free DNA% is elevated with pathogens that are risk factors for acute and chronic lung allograft injury. J Heart Lung Transplant.

[48] Wong L, Robert W, Dholakia S (2022). Medical diagnostic methods the evolution and innovation of donor-derived cell-free DNA testing in transplantation.

[49] Arcasoy SM, Berry G, Marboe CC, Tazelaar HD, Zamora MR, Wolters HJ (2011). Pathologic interpretation of transbronchial biopsy for acute rejection of lung allograft is highly variable. Am J Transplant.

[50] • Keller M, Sun J, Mutebi C, Shah P, Levine D, Aryal S, et al. Donor-derived cell-free DNA as a composite marker of acute lung allograft dysfunction in clinical care. J Heart and Lung Transplant. 2022;41(4):458–466. **Retrospective analysis from a multi-center collaboration assessing the clinical utility of a clinically-available dd-cfDNA assay for surveillance of lung transplant health during an early era of the COVID-19 pandemic, without the standard of care availability of surveillance bronchoscopy procedures.** 10.1016/j.healun.2021.12.009 35063338

[51] Huo J, Xu Y, Sheu T, Volk RJ, Shih YT (2019). Complication rates and downstream medical costs associated with invasive diagnostic procedures for lung abnormalities in the community setting. JAMA Intern Med.

[52] McWilliams TJ, Williams TJ, Whitford HM, Snell GI (2008). Surveillance bronchoscopy in lung transplant recipients: risk versus benefit. J Heart Lung Transplant.

[53] Levy L, Huszti E, Pal P, Tikkanen J, Ghany R, Keshavjee S (2021). The impact of inadequate (“AX”) transbronchial biopsies on post-lung transplant CLAD or death. Transplantation.

[54] Langelier C, Kalantar KL, Moazed F, Wilson MR, Crawford ED, Deiss T (2018). Integrating host response and unbiased microbe detection for lower respiratory tract infection diagnosis in critically ill adults. Proc Natl Acad Sci.

[55] Hogan CA, Yang S, Garner OB, Green DA, Gomez CA, Dien Bard J (2020). Clinical impact of metagenomic next-generation sequencing of plasma cell-free DNA for the diagnosis of infectious diseases: a multicenter retrospective cohort study. Clin Infect Dis.

[56] Lee RA, Dhaheri FA, Pollock NR, Sharma TS, Dekker JP (2020). Assessment of the clinical utility of plasma metagenomic next-generation sequencing in a pediatric hospital population. J Clin Microbiol.

[57] Wang Z, Gerstein M, Snyder M (2009). RNA-Seq: a revolutionary tool for transcriptomics. Nat Rev Genet.

[58] Emerson JB, Adams RI, Román CMB, Brooks B, Coil DA, Dahlhausen K (2017). Schrödinger’s microbes: tools for distinguishing the living from the dead in microbial ecosystems. Microbiome.

[59] Naccache SN, Federman S, Veeraraghavan N, Zaharia M, Lee D, Samayoa E (2014). A cloud-compatible bioinformatics pipeline for ultrarapid pathogen identification from next-generation sequencing of clinical samples. Genome Res.

[60] Moss J, Magenheim J, Neiman D, Zemmour H, Loyfer N, Korach A (2018). Comprehensive human cell-type methylation atlas reveals origins of circulating cell-free DNA in health and disease. Nat Commun.

[61] Andargie TE, Tsuji N, Seifuddin F, Jang MK, Yuen PS, Kong H (2021). Cell-free DNA maps COVID-19 tissue injury and risk of death and can cause tissue injury. JCI insight.

[62] Lehmann-Werman R, Neiman D, Zemmour H, Moss J, Magenheim J, Vaknin-Dembinsky A (2016). Identification of tissue-specific cell death using methylation patterns of circulating DNA. Proc Natl Acad Sci U S A.

[63] Gai W, Zhou Z, Agbor-Enoh S, Fan X, Lian S, Jiang P, et al. Applications of genetic-epigenetic tissue mapping for plasma DNA in prenatal testing, transplantation and oncology. Elife. 2021;10. 10.7554/eLife.64356 PMC7997656 33752803

[64] Tsuji N, Agbor-Enoh S (2021). Cell-free DNA beyond a biomarker for rejection: biological trigger of tissue injury and potential therapeutics. J Heart Lung Transplant.

[65] Suarez-Alvarez B, Baragano Raneros A, Ortega F, Lopez-Larrea C (2013). Epigenetic modulation of the immune function: a potential target for tolerance. Epigenetics.

[66] Zhu C, Xiang W, Li B, Wang Y, Feng S, Wang C (2021). DNA methylation modulates allograft survival and acute rejection after renal transplantation by regulating the mTOR pathway. Am J Transplant.

[67] Green ED, Watson JD, Collins FS (2015). Human Genome Project: twenty-five years of big biology. Nature.

